# A multivariate approach to amperometric free chlorine monitoring: calibration, interference compensation, and application in water distribution systems

**DOI:** 10.1039/d6ra07165a

**Published:** 2026-09-25

**Authors:** Guglielmo Emanuele Franceschi, Lisa Rita Magnaghi, Gian Paolo Quarta, Raffaela Biesuz

**Affiliations:** a Department of Chemistry, University of Pavia Viale Taramelli 12 Pavia PV 27100 Italy lisarita.magnaghi@unipv.it; b INSTM, Unità di Ricerca di Pavia Via G. Giusti 9 Firenze FI 50121 Italy; c Onyax – Soluzioni IoT Via P. Bertolini, 9/L Vigevano PV 27029 Italy

## Abstract

Reliable monitoring of Free Available Chlorine (FAC) in drinking water is essential for ensuring disinfection efficiency and detecting contamination events, yet *in situ* amperometric measurements remain strongly affected by matrix variability and long-term instability. In this work, we present an optimized amperometric protocol for FAC determination implemented in NEMO, a low-cost, battery-powered Online Water Quality Monitoring (OWQM) device designed for continuous operation in water distribution systems. The working potential was identified through cyclic voltammetry and subsequently optimized using a multivariate Design of Experiments (DoE) approach. To address the strong dependence of the amperometric signal on pH and conductivity, two Multiple Linear Regression calibration strategies were developed: an *in silico* calibration model (Approach A) and a direct multivariate model (Approach B). Both approaches were built on 125 calibration samples and validated on an independent test set, showing a consistent agreement with the reference DPD spectrophotometric method. Limits of detection were evaluated through four complementary procedures, confirming suitability for typical FAC levels in drinking water. Field deployment on six devices over two months demonstrated overall stable performance, limited hysteresis, and reliable tracking of temporal FAC variations. The proposed methodology enables robust semi-quantitative chlorine monitoring in real networks and represents a scalable solution for distributed early-warning systems in water quality management.

## Abstract 2

Multivariate amperometric sensing for free chlorine: optimized NEMO protocol compensates pH and conductivity effects, enabling stable, reagent‑free monitoring in real water networks.

## Graphical abstract

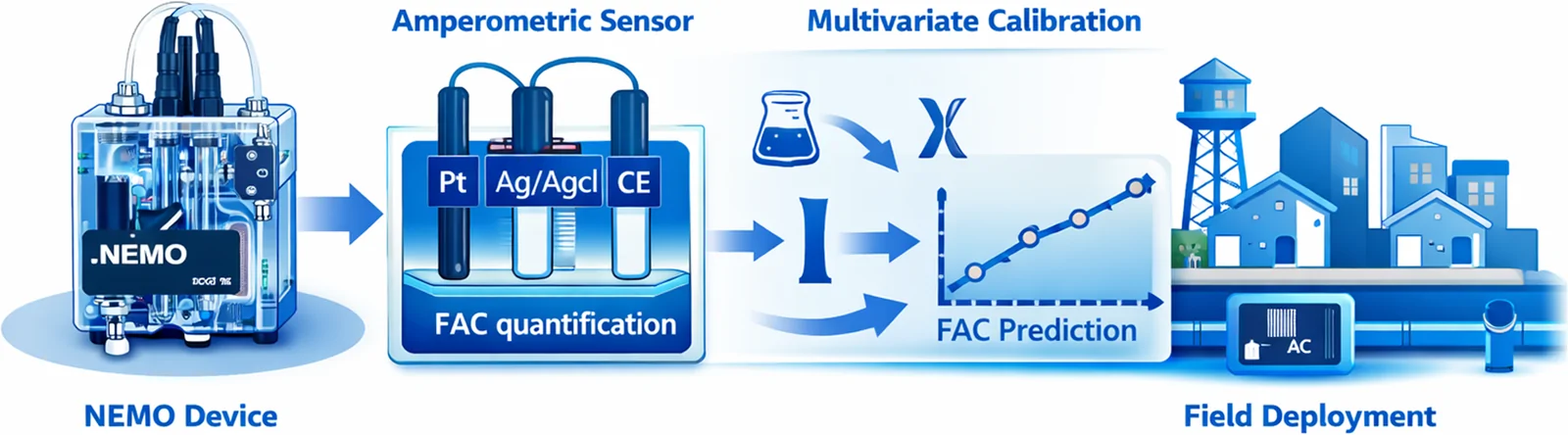

## 1 Introduction

In the 2000's, water-related issues in distribution systems represented a multifaceted challenge in which chemistry, data science, engineering, economics and sociology coexist and provide complementary viewpoints. An almost perfect agreement on actual risks and future goals has been established: (i) increasing demand, associated with population growth and urbanization,^1^ (ii) water shortage, mainly provoked by climate change and anthropogenic activities^2,3^ and further exacerbated by diffuse leakage events in aging and underfunded infrastructures,^1–3^ (iii) quality and security deterioration, due to anthropic, infrastructure-related and pollution-related contaminations^2,3^ and (iv) out-of-date policies and procedures in water management^2,3^ which undoubtedly represent the major contemporary water-related problems. For this reason, back in 2018, the Global Risk Report indicated that most high-risk issues (high-likelihood and high-impact) involve water supply.^4^ On the contrary, Sustainable Development Goals (SDGs) clearly state the overarching goal that the entire field should aim to achieve: improved water availability, sustainable management of water and sanitation for the entire population.^1^ Similarly, the United Nations state “The human right to water entitles everyone without discrimination to sufficient, safe, acceptable, physically accessible, and affordable water for personal and domestic use”.^1^

How to address the listed issues and, more importantly, how technological developments and digital transformation can support Water Distribution Systems (WDS), remain open questions both in the academic and industrial landscape: on one hand, the urgent need to modernize and re-imagine supply networks and technologies to meet today's needs is well-known and beyond doubt but the required time and costs strongly limit the investments in this direction.^3^ On the other hand, retrofitting existing WDS by adding smart components for improved network monitoring in terms of supply, consumption, leakage, and quality,^3^ seems to be a more accessible strategy in terms of time and cost and ensures immediate benefits to all parties in the hydrosocial contract, as nicely defined by C. Hoolohan and coworkers.^2^

Low-cost and *in situ* technologies, generally called Online Water Quality Monitoring (OWQM) devices,^5^ can significantly contribute to several water-related issues. Firstly, real-time assessment of water quality ensures appropriate and timely response to accidental or deliberate contamination, in contrast to laboratory-based methods that involve a significant delay,^6^ and enhances citizens' trust and their participation in water governance.^2^ Additionally, decentralized and localized monitoring within WDS can assist urgent maintenance interventions by identifying the network fragments more prone to infrastructure-related problems and enhance the reliability and consumption monitoring of localized distribution points, such as Water ATMs.^2^ It is evident that devices designed for such a critical task must comply with very stringent requirements: analytical reliability and robustness, low power consumption and maintenance frequency, remote control, data communication systems and, finally, minimization of installation and operational costs.^7^

Analytical reliability plays a paramount role in the development of OWQM devices, especially when aiming at their application as Early Warning Systems (EWS), as precisely explained by M. V. Storey and coauthors: EWS should guarantee timely detection of low-probability/high-impact contamination by distinguishing between normal fluctuations, contamination events, and consistent water-quality variations.^6^ With this application in mind, robustness and a limited occurrence of false positives or false negatives must be prioritized over enhanced exactness, comparable to conventional techniques.^6^

Following this direction, we focused our investigation on assessing Free Available Chlorine (FAC) in drinking water, and optimized the amperometric protocol embedded in the OWQM device NEMO, developed, and manufactured by Onyax srl (Vigevano, Italy).^7–9^ This device, recently presented in the literature for conductivity quantification,^7–9^ is here investigated for FAC detection. Assessing the concentration of free chlorine in the water is essential for monitoring and detecting the presence of contaminants, as this molecule is one of the main disinfectants in public water supplies yet, quite surprisingly, FAC is not included in the Water Quality Index, originally proposed in the '60s.^5^ In fact, insufficient chlorine dosing may result in incomplete disinfection while excessive dosing may result in unpleasant odour and taste, increase the levels of halogenated compounds and increase both economic and environmental impact.

The well-established importance of free chlorine in ensuring water quality and potability is outweighed by the challenging nature of *in situ* FAC assessment; consequently, the development of reliable free-chlorine sensors in water has progressed gradually and should not be considered fully established.^5^ Free Available Chlorine (FAC) can be quantified through a variety of analytical strategies, each characterized by specific operational constraints that become critical in online monitoring.^5^ Colorimetric DPD-based methods remain the regulatory standard, but their reliance on reagents, manual handling and batch-wise operation limits applicability in continuous OWQM deployments. Recent electrochemical approaches have therefore focused on reagent-free amperometric detection, yet the literature consistently highlights several unresolved bottlenecks that hinder robust *in situ* quantification. Among the different FAC sensing strategies, amperometric sensors undoubtedly represent the most common one, resulting in a heterogeneous landscape in literature: proposed devices can be distinguished according to electrode materials, target concentration range, batch *vs.* in-flow applicability, short- *vs.* long-term performance, hysteresis susceptibility and robustness against interferents, mainly nitrates, sulphates, and bicarbonates.^10,11^ Consequently, measurement parameters and experimental setups vary widely among the proposed solutions, ranging from early lab-scale prototypes^12–14^ to devices suitable for online implementation.^10,15^

Focusing on the most recent literature, a first class of studies demonstrates that electrode materials and surface engineering strongly determine sensitivity, stability and susceptibility to matrix effects. For example, Prussian Blue-based architectures enable low-potential electrocatalytic reduction of HOCl but require careful control of deposition, membrane integrity and long-term stability to avoid drift and hysteresis. This is evident both in microfluidic NFC-powered systems,^16^ where the PB-GI@CNT composite shows excellent electrocatalytic performance but still requires controlled flow, stable pH and periodic recalibration, and in low-cost screen-printed electrodes for decentralized reuse systems,^17^ where reproducibility across sensors depends critically on PB film thickness and deposition chemistry. Even more advanced PB-coated SPCEs tested over one-month continuous operation exhibit sensitivity decay and require stabilization periods of several days, confirming that long-term amperometric monitoring remains challenging.^18^

Other material-driven approaches, such as Multi-Walled Carbon Nanotube (MWCNT)-modified TPU electrodes^19^ or Metal–Organic Framework (MOF)-based Fe_3_O_4_–Cu-BTC composites,^20^ achieve high sensitivity and wide linear ranges, but they are typically validated under controlled laboratory conditions and do not address the variability of drinking-water matrices. Across all these systems, pH, conductivity, temperature and dissolved oxygen emerge as dominant interferents. Temperature-dependent shifts in HOCl/OCl^−^ speciation and diffusion coefficients have been quantified in detail by Yamanouchi *et al.*,^21^ who show that even modest thermal fluctuations produce non-uniform distortions in voltametric curves, complicating calibration and degrading accuracy. Their work also demonstrates that simple univariate calibration is insufficient, and that multivariate or machine-learning models can partially compensate for matrix-dependent distortions, but at the cost of increased model complexity, risk of overfitting, and limited transferability across sensors or networks.

Taken together, these studies reveal that suitable calibration strategies for amperometric FAC sensor that simultaneously ensure low-cost, transferability across devices, robustness to pH and conductivity variability, and suitability for long-term autonomous operation in real distribution networks represent a challenging task to be addressed.^22^ In this scenario, we focused on amperometric FAC assessment by means of commercial probes embedded in the NEMO device, thus ensuring prompt scalability toward in-field application, achieved by directly installing the device in WDSs. First, measurement parameters are optimized following a multivariate approach, using Design of Experiments (DoE) tools; then, robustness against common interferents in tap water analysis, namely matrix pH and conductivity (*χ*), is addressed by developing multivariate calibration models able to account for their impact in the quantification process. The proposed methodology is validated by comparison with the reference methodology, *i.e.* spectrophotometric quantification by *N*,*N*-diethyl-*p*-phenylenediamine (DPD), and a panel of statistical approaches is evaluated in LOD assessment for pH and *χ*-dependent models. Lastly, in-field application is tested by implementing the FAC quantification methods in previously installed NEMO devices and monitoring the current values and corresponding FAC concentrations for two months. This preliminary analysis allows us to address the hysteresis issue and long-term performance, two of the most critical requirements of OWQM devices.

## 2 Materials and methods

### 2.1 Experimental set up description

NEMO is a portable chemical micro-laboratory with an integrated battery and remote transmission, designed and manufactured by Onyax srl (Vigevano, PV, Italy); its small size (120 × 150 × 60 mm) allows NEMO to be installed directly in the housing or in the control points of the WDS, as shown in Fig. S1.^7^ To better simulate real-case scenario, NEMO device has been connected to a distribution system-simulation apparatus that includes a peristaltic pump, ensuring a constant water flow up to 30 L h^−1^, tanks of different capacities and a closed system for water circulation.^7^

NEMO device includes a three-electrodes electrochemical cell, composed of a platinum Working Electrode (WE), an Ag/AgCl Reference Electrode (RE) positioned within the KCl 3M electrolyte, placed in contact with the sample through a permeable diaphragm and an INOX steel Counter Electrode (CE), all embedded in the Plexiglas body of NEMO. In this configuration, the setup allows performing amperometry for FAC determination by applying a potential between WE and RE, while resulting current to flow between WE and CE. Additionally, a commercial potentiostat is included in the NEMO device and the operational parameters can be tuned directly within NEMO microcontroller firmware to ensure automated measurements while reducing electrical noise by replacing external cables with the microsoldered circuitry.

### 2.2 FAC determination optimization

#### 2.2.1 Suitable potential range identification by cyclic voltammetry (CV)

To identify the suitable potential range considering acid–base behaviour of chlorine species, buffered solutions (pH = 3.5, 5.5, 7.5, and 9.5) with increasing free chlorine concentration (0–5 mg L^−1^) are analysed using cyclic voltammetry with Metrohm Autolab B.V. (Autolab, The Netherlands) in the potential range −0.4 to 0.6 V. To better mimic the experimental conditions, CV is performed using a platinum WE, Ag/AgCl RE and an INOX steel CE; solution-preparation details are reported in Table S1.

#### 2.2.2 Amperometry settings optimization by DoE

The NEMO hardware allows tuning of various input parameters for amperometry, such as the potential value for WE and RE, the current range of interest and the amplification gain, while other values depend on the actual chipset, such as calibration resistance (*R*_calibration_ = 10 kΩ) or additional resistance (*R*_load_ = 100 Ω). In this case, the current range and amplification gain are set to their minimum values (10 µA and 1) to reduce battery consumption in the in-field prototype, while the electrode potentials are optimized by means of a face-centred design. All calculations are performed using the CAT software.^23^

Instead of individually considering the potential value for each electrode as included in the NEMO firmware, the potential difference (*x*_1_) and the central potential value (*x*_2_) are computed to better describe the system, highlighting the correlation between the electrode potentials, and to facilitate the interpretation of the results, generally expressed in terms of potential differences rather than the potential value at each electrode. The selected levels for each variable are described in Table 1.

**Table 1:** Variables selection and levels definition for settings optimization by DoE

Variable	Abbreviation		Lower level	Central level	Upper level
Potential difference	Δ*V*	*x*_1_	−300 mV	−200 mV	−100 mV
Central potential value	Mean *V*	*x*_2_	500 mV	700 mV	900 mV

The model equation, reported in eqn (1), includes the linear, interaction, and quadratic terms for all the variables, while the list of training experiments, including a triplicate of the [0,0] point, is reported in Table S2. The coefficients are computed for two independent responses: average current over a 20 s potential input for tap water before (*y*_1_, blank I) and after the addition of 0.5 mg L^−1^ FAC addition (*y*_2_, 0.5 mg L^−1^ FAC I). This potential-input duration was previously shown to represent a good compromise.^24^ The obtained response surfaces are jointly analysed to identify the best compromise between minimizing the blank signal (*y*_1_) and maximizing the analyte signal (*y*_2_).

Eqn (1): Model equation for settings optimization:

1 *y* = *b*_0_ + *b*_1_*x*_1_ + *b*_2_*x*_2_ + *b*_12_*x*_1_*x*_2_ + *b*_11_*x*_1_^2^ + *b*_22_*x*_2_^2^

### 2.3 pH and conductivity-dependent calibration models development for FAC amperometric detection

Once FAC measurement is optimized, the interference from water pH and conductivity on the amperometric signal must be addressed to enable in-field applications, in which tap water pH and conductivity (*χ*) can be measured simultaneously but cannot be controlled. Indeed, the calibration model itself must consider not only the analyte concentration but also matrix characteristics: to address this issue, calibration samples are prepared under different pH and *χ* conditions, and two different approaches are proposed here to include matrix effects in the calibration model.

#### 2.3.1 Calibration, blank and test samples preparation

The calibration samples are prepared following a typical DoE methodology: the typical ranges for pH and *χ* in tap water, ranging between 6 and 8 pH units and between 25 and 600 µS cm^−1^, are divided into five levels. Bottled waters samples exhibiting the required *χ* value (15, 135, 225, 420 and 550 µS cm^−1^) are purchased from a local supermarket, the pH is adjusted to obtain the defined pH values for each bottled water by adding diluted HCl or NaOH, and the final *χ* is measured. This framework results in 25 combinations of pH and *χ* (five pH levels *x* five *χ* levels) to be included in the calibration model, as described in Table 2.

**Table 2:** Calibration samples for pH and conductivity-dependent calibration

Bottled water	Combination	Final pH	Final *χ* after pH adjustment
A	A1	6.0	50.0 µS cm^−1^
	A2	6.5	45.0 µS cm^−1^
	A3	7.0	37.5 µS cm^−1^
	A4	7.5	33.5 µS cm^−1^
	A5	8.0	25 µS cm^−1^
B	B1	6.0	141.5 µS cm^−1^
	B2	6.5	136.0 µS cm^−1^
	B3	7.0	138.0 µS cm^−1^
	B4	7.5	140.0 µS cm^−1^
	B5	8.0	141.5 µS cm^−1^
C	C1	6.0	350.0 µS cm^−1^
	C2	6.5	300.0 µS cm^−1^
	C3	7.0	275.0 µS cm^−1^
	C4	7.5	241.0 µS cm^−1^
	C5	8.0	235.0 µS cm^−1^
D	D1	6.0	442.0 µS cm^−1^
	D2	6.5	436.0 µS cm^−1^
	D3	7.0	426.0 µS cm^−1^
	D4	7.5	417.0 µS cm^−1^
	D5	8.0	423.0 µS cm^−1^
E	E1	6.0	614.0 µS cm^−1^
	E2	6.5	603.0 µS cm^−1^
	E3	7.0	593.0 µS cm^−1^
	E4	7.5	574.0 µS cm^−1^
	E5	8.0	585.0 µS cm^−1^

For each pH–*χ* combination, five solutions are prepared in a FAC concentration range of 0.06 to 0.75 mg L^−1^, by adding defined volumes of sodium hypochlorite solution 2.5%, resulting in a total of 125 training samples. Each sample is flowed through the distribution simulation system for one hour, to ensure system equilibration, and automatically measured twice using the NEMO microcontroller firmware.

Blank samples are also prepared in duplicate for each pH–*χ* combination without adding sodium hypochlorite solution and are used to evaluate the Limit of Detection (LOD), while the test set includes the same B-E bottled water at their native pH (bottled water A is excluded since the native conductivity is outside the limits of the model), fortified with the same FAC concentrations used for the calibration set, between 0.06 and 0.75 mg L^−1^ (Table S3).

#### 2.3.2 Calibration models development

Two different approaches are tested to develop a fully automated pH- and *χ*-dependent calibration model to be directly included in NEMO microcontroller firmware for in-field applications, both relying on Multiple Linear Regression (MLR) and referred to here as “Approach A” and “Approach B”.

Approach A involves two steps: in the first step, the calibration samples are employed to build a MLR model able to calculate the current value for each pH–*χ*–[FAC] triplet, setting the domain limits at 6–8 pH units, 25–600 µS cm^−1^ for *χ* and 0.06–0.75 mg L^−1^ for free chlorine concentration and coding the input values within the −1 to +1 range (eqn (2a)). In the second step, given the actual values of pH and *χ* measured in-field, an *in silico* calibration is performed by predicting the current values for calibration standards (*I*^pred^), equal in number and concentration to those in the training set, and computing the *in silico* regression line used to estimate free chlorine from the experimental current value.

Eqn (2): *In silico* calibration protocol for free chlorine quantification according to Approach A:

2a *I* (µA) = *α*_0_ + *α*_pH_ pH + *α*_*χ*_*χ* + *α*_FAC_ FAC + *α*_pH,*χ*_ pH *χ* + *α*_pH,FAC_ pH FAC + *α*_*χ*,FAC_*χ* FAC + *α*_pH,pH_ pH^2^ + *α*_*χ*,*χ*_*χ*^2^ + *α*_FAC,FAC_ FAC^2^

2b
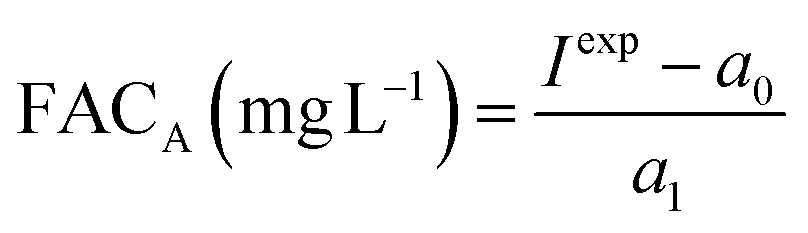
where *a*_0_ and *a*_1_ are computed by linear regression on predicted *I* for actual pH and *χ*

Approach B involves a single step: the calibration samples are employed to build a MLR model capable of directly calculating free chlorine concentration, expressed in mg L^−1^, from pH, *χ*, and current value. In this case, a general calibration equation, given in eqn (3), is implemented in the firmware and the measured parameters are used to directly estimate the analyte concentration.

Eqn (3): General calibration equation for free chlorine quantification:3
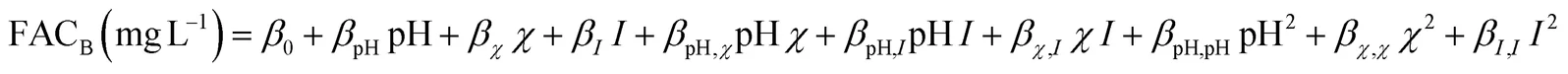


#### 2.3.3 Calibration models evaluation by test set prediction

Both approaches, A and B, respectively, are validated by comparing the experimental and predicted values for the test set samples. For Approach A, the comparison is established between experimental and predicted I values, for each bottled water (B-E) and FAC concentration; for Approach B, the same comparison is performed directly on FAC concentration, expressed in mg L^−1^.

The models are accepted when the Root Mean Square Error in Prediction (RMSEP), computed as reported in the literature,^7,25^ is not significantly higher than RMSE in cross-validation (RMSECV). This workflow is typical of chemometric regression models, but here is justified for the large abundance of degrees of freedom and the large number of test samples.

### 2.4 Figures of merit calculation for pH and conductivity-dependent calibration models

#### 2.4.1 Validation by comparison with reference methodology

The developed pH- and conductivity-dependent calibration models for FAC amperometric quantification are validated by comparison with the reference methodology, *i.e.* spectrophotometric quantification by *N*,*N*-diethyl-*p*-phenylenediamine (DPD), following the procedure described in *Standard Methods for the Examination of Water and Wastewater*.^26^

A 100 mL solution at increasing FAC concentration (0.06–0.75 mg L^−1^) is prepared as follows: defined volumes of 2.5% sodium hypochlorite solution are diluted to around 80 mL, then 1 mL H_2_SO_4_ 1 M is added to the solution and, after one minute, the solution is neutralized with 1 mL of 2 M NaOH and diluted to the final volume. A 1 L phosphate-buffer stock solution is prepared by dissolving 60.5 g Na_2_HPO_4_·12H_2_O, 46.0 g KH_2_PO_4_ and 0.80 g EDTA disodium salt and then equilibrated at pH 6.5. A 1 L DPD-reagent stock solution is prepared by adding 2 mL concentrated H_2_SO_4_ and 0.20 g EDTA disodium salt to 250 mL H_2_O; after homogenization, 1.10 g anhydrous DPD is then dissolved, and the solution is diluted to the final volume. In a flask, 5 mL phosphate-buffer solution and 5 mL DPD-reagent solution are mixed for 45 s before adding 100 mL FAC solution at the defined concentration; the spectrophotometric measurement must be performed within two minutes and absorbance at 515 nm is extracted by applying a three-point baseline correction (*λ*_1_ = 431 nm, *λ*_2_ = 597 nm, *λ*_peak_ = 515 nm) and used to build the regression line.

The procedure is applied for each matrix in the test set, B-E bottled water at their native pH values, to build pH- and *χ*-dependent regression lines for spectrophotometric FAC detection and, lastly, a sample fortified with a 0.38 mg L^−1^ FAC is prepared and analysed in duplicate both by amperometry (Approaches A and B) and the spectrophotometric method to assess the models’ accuracy.

#### 2.4.2 Limit of detection (LOD) estimation

Approaches A and B are compared in terms of sensitivity by computing an LOD range for each pH–*χ* combination, according to four different procedures: as already discussed for PLS models,^7,27^ a multivariate regression model cannot be associated with a single LOD value but a LOD range should be computed, in order to take into account matrix effects and model leverage.

Procedure 1 defines LOD on the average signal of experimental blank samples (*I*^exp^_b_) and standard deviation (*s*_b_), and the corresponding significant signal *I*_S_, according to the eqn (4a).^28^ For both approaches, LOD_1_ is defined as the analyte concentration corresponding to a signal equal to *I*_s_, although the calculation is slightly different, as reported in eqn (4b) and (4c).

Eqn (4): LOD definition from blank samples for both approach A (LOD_A1_) and B (LOD_B1_):

4a *I*_S,A_ = *I*^exp^_b_ + 2 × *t*_*α*,*ν*_ × *s*^exp^_b_

where *α* = 5% and *ν* = no. of blank samples -1.4b
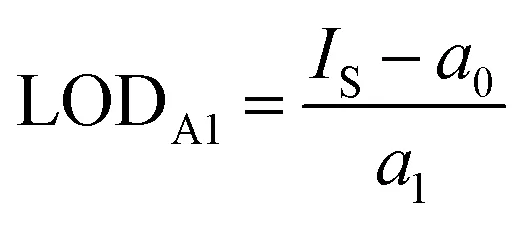
where LOD_A1_, *a*_0_ and *a*_1_ depend on actual pH and *χ*

4c LOD_B1_ = *β*_0_ + *β*_pH_ pH + *β*_*χ*_*χ* + *β*_*I*_*I*_S,A_ + *β*_pH,*χ*_ pH *χ* + *β*_pH,I_ pH *I*_S,A_ + *β*_*χ*,*I*_*χ I*_*S*_ + *β*_pH,pH_ pH^2^ + *β*_*χ*,*χ*_*χ*^2^ + *β*_*I*,*I*_*I*_S,A_^2^

Procedure 2 computes instead significant signal *I*_s_ by exploiting the calculated blank-sample signal, rather than the experimental one, together with the standard deviation of calibration-sample residuals (*s*_res_). The latter parameter can be computed similarly for both approaches, while the calculated blank signal corresponds to *a*_0_ for traditional calibration lines in Approach A (eqn (5a)) but does not correspond to *β*_0_ in approach B. In fact, the use of coded input values results in a different physical significance of this term, no longer referring to the absence of the analyte but to the centre of the experimental domain. For this reason, the blank signal is calculated for each pH–*χ* combination by considering FAC = 0 in eqn (3) (eqn (5b)) and selecting the second-degree equation solution nearest to the tested experimental domain, coded within the −1 to +1 range, always corresponding to the positive one (eqn (5c) and (5d)). Applying this procedure, the resulting LOD values are computed as reported in eqn (5e) and (5f), for Approaches A and B respectively.

Eqn (5): LOD definition from s_res_ for both approach A (LOD_A2_) and B (LOD_B2_):

5a *I*_S,A_ = *a*_0_ + 2 × *t*_*α*,*ν*_ × *s*_res_

where *α* = 5% and *ν* = regression line's degrees of freedom (dof)

5b *β*_I,I_*I*^2^ + (*β*_*I*_ + *β*_pH,*I*_ pH + *β*_*χ*,*I*_*χ*)*I* + *β*_0_ + *β*_pH_ pH + *β*_*χ*_*χ* + *β*_pH,*χ*_ pH *χ* + *β*_pH,pH_ pH^2^ + *β*_*χ*,*χ*_*χ*^2^ = 0

5c
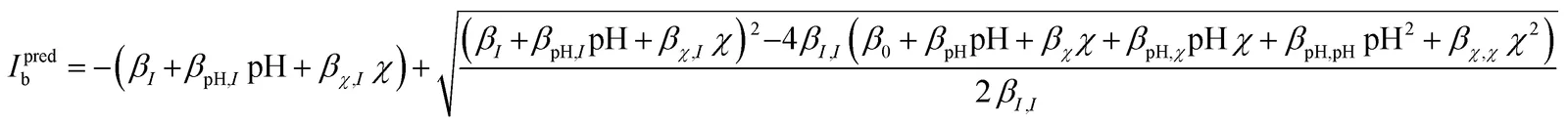

5d *I*_S,B_ = *I*^pred^_b_ + 2 × *t*_*α*,*ν*_ × *s*_res_

where *α* = 5% and *ν*= MLR model dof5e
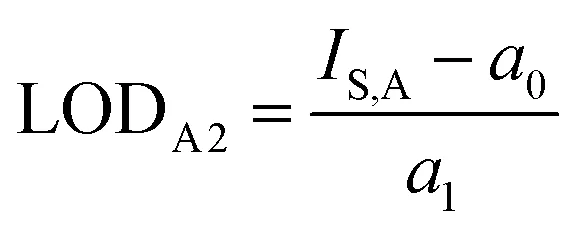
where LOD_A2_, *a*_0_ and *a*_1_ depend on actual pH and *χ*.

5f LOD_B2_ = *β*_0_ + *β*_pH_ pH + *β*_*χ*_*χ* + *β*_*I*_*I*_S,B_ + *β*_pH,*χ*_ pH *χ* + *β*_pH,*I*_ pH *I*_S,B_ + *β*_*χ*,*I*_*χ I*_S,B_ + *β*_pH,pH_ pH^2^ + *β*_*χ*,*χ*_*χ*^2^ + *β*_*I*,*I*_*I*_S,B_^2^

Lastly, Procedures 3 and 4 rely on the iterative Haubax–Vos methodology,^28^ which can be summarised results in: LOD is the concentration whose signal significantly exceeds noise. From a mathematical point of view, LOD Lower Limit (LL_LOD_) of confidence interval (*α* = 5% CL and *ν*= regression model's dof) is equal to blank Upper Limit (UL_b_) of confidence interval. For Approach A, blank UL_b_ is computed as reported in eqn (6a), replacing *C*_b_ with the concentration computed as in eqn (6b) and the standard deviation in eqn (6c); LL_LOD_ is computed with the same set of equations, replacing the + sign with − and treating eqn (6c) as a function of concentration (eqn (6d)). Putting together the eqn (6a)–(6d), the iterative LOD definition in eqn (6e) is obtained, which shows a satisfactory convergence in a few cycles. Procedure 3 and 4 differ according to *I*_b_ in eqn (6b) and (6c): the former uses experimental blanks as in Procedure 1 while the latter uses the intercept as in Procedure 2.

Eqn (6): LOD definition from Haubax–Vos methodology for approach A and B, starting from experimental blanks (Procedure 3) or computed values (Procedure 4):

6a UL_b_ = *C*_b_ + *t*_*α*,*ν*_ × *s*_*C*_b__

where *α* = 5% and *ν* = regression line's degrees of freedom (dof)6b
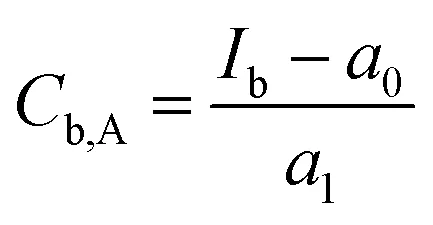
6c
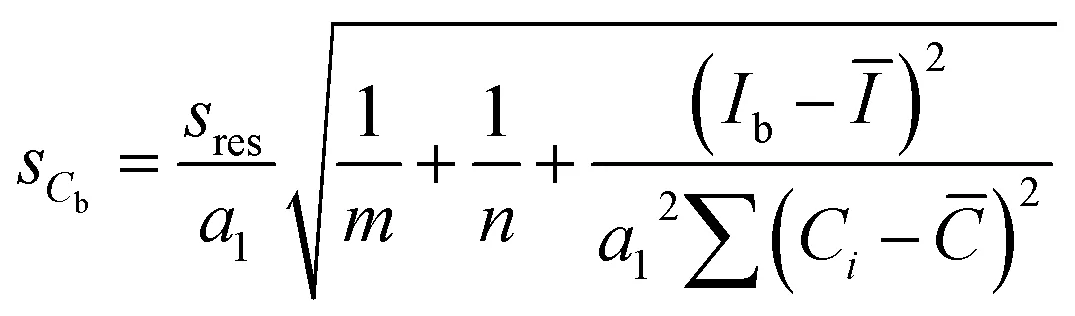
where *m* = calibration standards (5), *n* = blanks (2), *a*_0_ and *a*_1_ depend on actual pH and *χ*.

6d *I*_LOD_ = LOD × *a*_1_ + *a*_0_; *Ī* = *C̄* × *a*_1_ + *a*_0_

6e
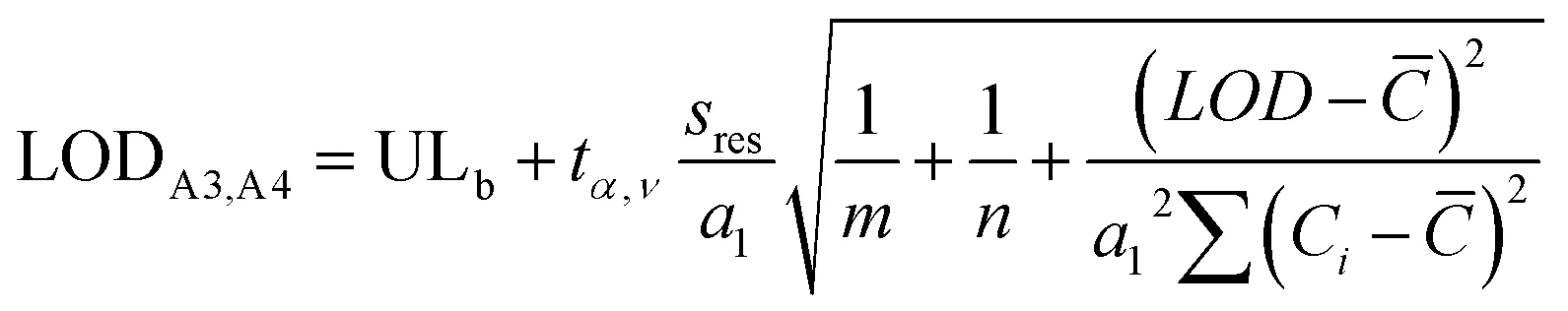

6f LOD_B3,B4_ = UL_b_ + *t*_*α*,*ν*_ × *s*_res_ × leverage (pH,*χ*,*I*_LOD_)

where *α* = 5% and *ν*= MLR model dof.

6g *β*_*I*,*I*_*I*^2^ +(*β*_*I*_ + *β*_pH,*I*_ pH + *β*_*χ*,*I*_*χ*)*I* + *β*_0_ + *β*_pH_ pH + *β*_*χ*_*χ* + *β*_pH,*χ*_ pH *χ* + *β*_pH,pH_ pH^2^ + *β*_*χ*,*χ*_*χ*^2^ = LOD

6h
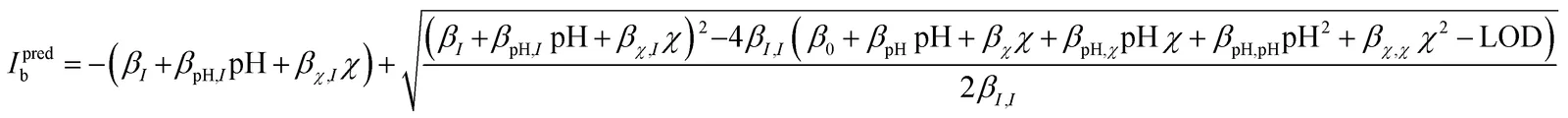


The same approach is applied also to Approach B with slight modifications: for UL_b_ calculation, *C*_b_ is computed by using *I*_b_ in model equation (eqn (3)), and the respective confidence interval also considers the leverage value for pH–*χ*–*I* triplet. Also in this case, experimental blanks or computed equivalents are used in Procedure 3 and 4, respectively. The LOD is thus defined with a similar approach, considering the leverage value for pH–*χ*–*I* triplet (eqn (6f)), where LOD is computed by considering FAC = LOD in eqn (3), as shown in eqn (6g) and (6h). Also in this case, the iterative definition shows a convergence in a few steps, for both Procedure 3 and 4.

### 2.5 In field validation for automated FAC assessment using optimized NEMO

Once lab-scale calibration-model development and validation are completed, described in the previous sections, both Approach A and B are implemented in the firmware on board NEMO devices, installed in WDSs. For this first in-field validation, six NEMO devices are selected, all installed in a 6 km-wide area in the Northern part of Italy, and data are collected for two months to assess the stability and reliability of the measurement protocol.

## 3 Results and discussion

In the present manuscript amperometric FAC detection in tap-water monitoring is presented: the optimization is directly conducted using NEMO, a portable device with integrated battery and remote transmission, designed and manufactured by Onyax srl (Vigevano, PV, Italy) to be directly installed in the housing or in the control points of WDSs. It goes without saying that the accuracy of the proposed methodology cannot be directly compared to traditional methods, performed in established laboratories using advanced instruments, trained personnel and validated procedures. Nevertheless, the NEMO device has the potential to continuously perform FAC semi-quantitative assessments, which are particularly relevant for an EWS application, as hinted in the introduction.

### 3.1 Identification of optimal potential range for FAC detection

Despite the semi-quantitative nature of the proposed methodology, or probably due to it, optimization of the working parameters is essential to achieve the best results from the simple and low-cost device employed. The first step in the optimization is the identification of a suitable working-potential range: to fulfil this purpose, cyclic voltammetry is applied in a potential range from −0.4 V to 0.6 V.

This task is critical for several reasons: from a chemical standpoint, reactions involving chlorine must be considered. In fact, chlorine is added to drinking water either in gaseous form (Cl_2_) or as a hypochlorite-containing salt, typically sodium hypochlorite (NaClO); when in contact with water, chlorine gas disproportionates into hydrochloric acid and HClO, the latter dissociates to form ClO^−^.^11,13,24,29^ Similarly, hypochlorite ion speciates in a pH-dependent fashion to form the oxidizing agents HClO and ClO^−^. The equilibrium constants for these reactions are around 3.4 and 7.5,^29^ respectively; thus the overall pH-dependent speciation can be summarized as shown in Fig. S2. In the typical pH range of tap water, between 6 and 8, only HClO and ClO^−^ are present in significant but variable amounts; for this reason, the definition of free chlorine or Free Available Chlorine (FAC) has been established as the sum of these two concentrations, typically expressed in parts per million (ppm, mg L^−1^).^14,29^

Other critical issues are more related to electrochemical cell shape, dimensions and electrode materials: literature examples include a wide variety of electrochemical setups and a broad panorama of working potentials, making an *a priori* definition of the optimal value unreliable.^10,30^

To consider all these aspects, CV measurements are conducted using a platinum WE, Ag/AgCl RE and INOX steel CE, to mimic the electrode materials employed in NEMO, across a pH range 3.5–9.5, to explore the different speciation scenarios, at increasing FAC concentrations (0, 2.5 and 5 mg L^−1^). The results are summarized in Fig. 1. The cathodic response is strongly dependent on the solution pH, which dictates the speciation of free chlorine, as discussed above: in acidic conditions (Fig. 1a and b), where hypochlorous acid (HClO) is the predominant species, a cathodic peak is observed together with a correlation between peak current and FAC concentration. As the pH approaches the p*K*_a_ (Fig. 1c, pH 7.5), the onset of the reduction potential shifts slightly, and a decrease in peak current is observed. This transition reflects the decreasing mole fraction of HClO in favor of the less-electroactive ClO^−^ species.

**Fig. 1:**
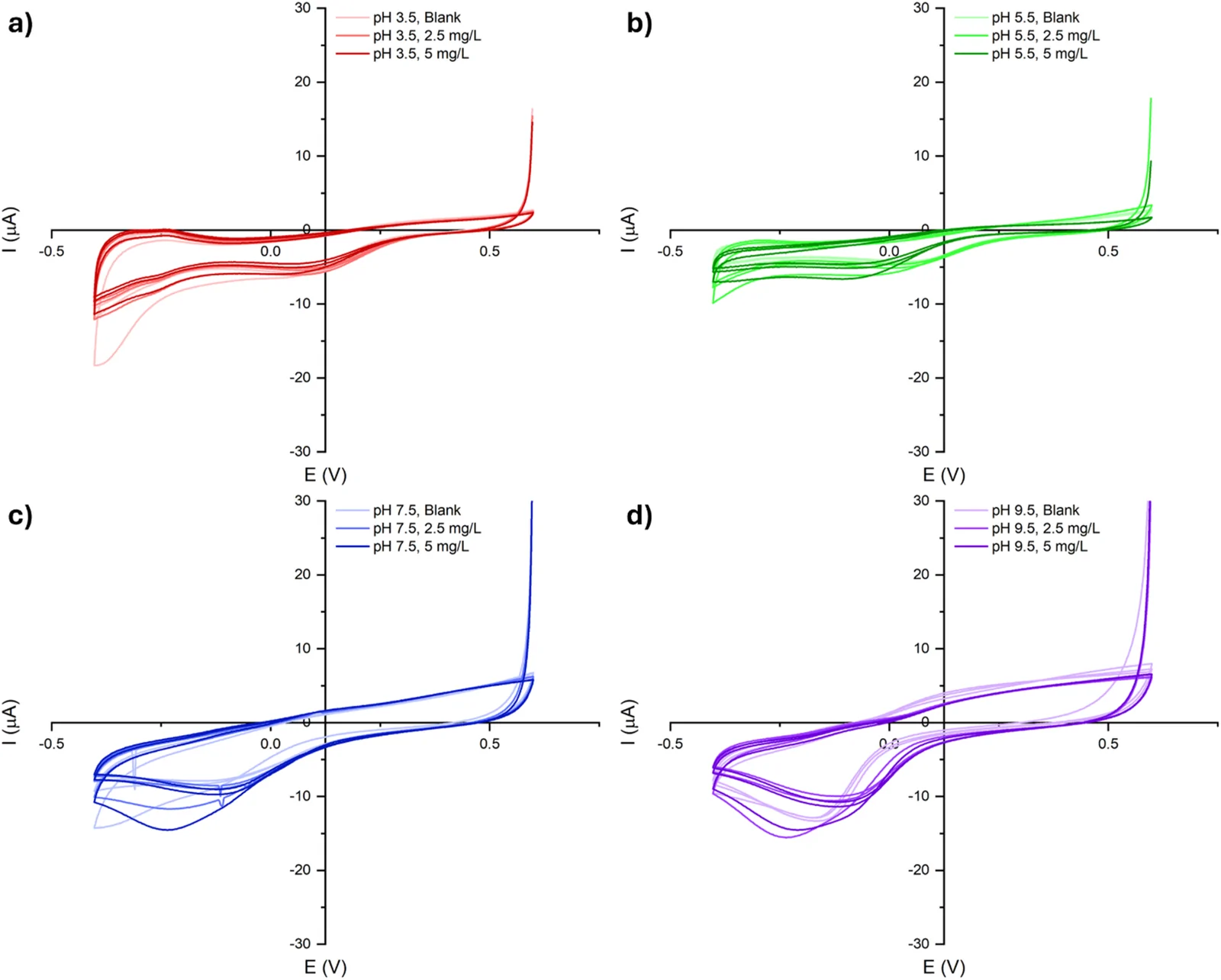
Cyclic voltammetry analysis for solutions at pH 3.5 (a), 5.5 (b), 7.5 (c) and 9.5 (d) at increasing FAC concentration (0, 2.5 and 5 mg L^−1^).

Under alkaline conditions (Fig. 1d), where ClO^−^ dominates, the reduction onset shifts toward more negative potentials. While this results in a less defined peak in the voltammetric profile compared to acidic conditions, the absolute current remains significant. However, it is important to note that the higher current values observed in the cathodic region are partially due to the overlapping contribution of the Oxygen Reduction Reaction (ORR), with a stronger effect at extreme pH values (Fig. 1a and d).

Despite the maximum theoretical sensitivity and peak definition being observed at pH 5.5, the NEMO device is designed for direct implementation in WDSs where pH modulation is not feasible. Consequently, the challenge lies in identifying a working potential that remains robust within the typical tap water pH range (6–8).

While some literature suggests operating at positive potentials to ensure maximum selectivity against oxygen,^12,13,26,30^ our experimental evidence indicates that the most stable analytical response in real-world water matrices is achieved between −0.1 V and −0.3 V *vs.* Ag/AgCl. This choice might be justified by three main factors: (i) at these more negative potentials, the system operates on the diffusion-limited plateau for both HClO and ClO^−^, minimizing the impact of minor pH fluctuations on the total current, as both species are effectively reduced at the electrode surface; (ii) maintaining a cathodic potential prevents the formation of passivating platinum-oxide layers, which typically occur at more positive potentials and can lead to signal drift over time; (iii) by targeting the plateau rather than the peak onset, the sensor becomes less sensitive to small variations in the reference electrode potential, leading to enhanced long-term reproducibility in continuous monitoring.

These observations highlight the common gap between idealized electrochemical models and the practical requirements of on-site sensing. While CV offers a roadmap for potential selection, the final choice of operating parameters must prioritize the resilience of the device under real-world fluctuations over strict adherence to theoretical maxima. For this reason, a candidate range between −300 mV and −100 mV is preliminarily identified and included as a variable in the following DoE approach. Such a range is in good agreement with literature case studies suggesting avoiding extreme potentials and preferring low-potential ranges.^24^

### 3.2 Amperometric settings optimization in NEMO device

The following step includes the optimization of amperometric settings by applying a DoE approach, as described in Section 2.2.2. Having well in mind the gap between lab-scale and in-field devices, NEMO firmware allows tuning of the conditions used for amperometric FAC detection by setting the actual potential value for the WE and CE electrodes. To better describe the system and make interpretation of the results more straightforward, we set the potential difference (Δ*V*, *x*_1_) and the central potential value (mean *V*, *x*_2_) as variables and we considered average currents over a 20 s potential input, for blank tap-water samples (*y*_1_) and after 0.5 mg L^−1^ FAC addition (*y*_2_) as responses.

The face-centred design, applied as described in Section 2.2.2, allows computing linear, quadratic and two-terms interaction coefficients, depicted in Fig. 2 for the two responses, while model statistics are summarized in Table S4. For the first response, *y*_1_ (blank-sample current), both linear terms are significant at a 95% confidence level while, for the second response, *y*_2_ (0.5 mg L^−1^ FAC current), only the mean-potential value is significant.

**Fig. 2:**
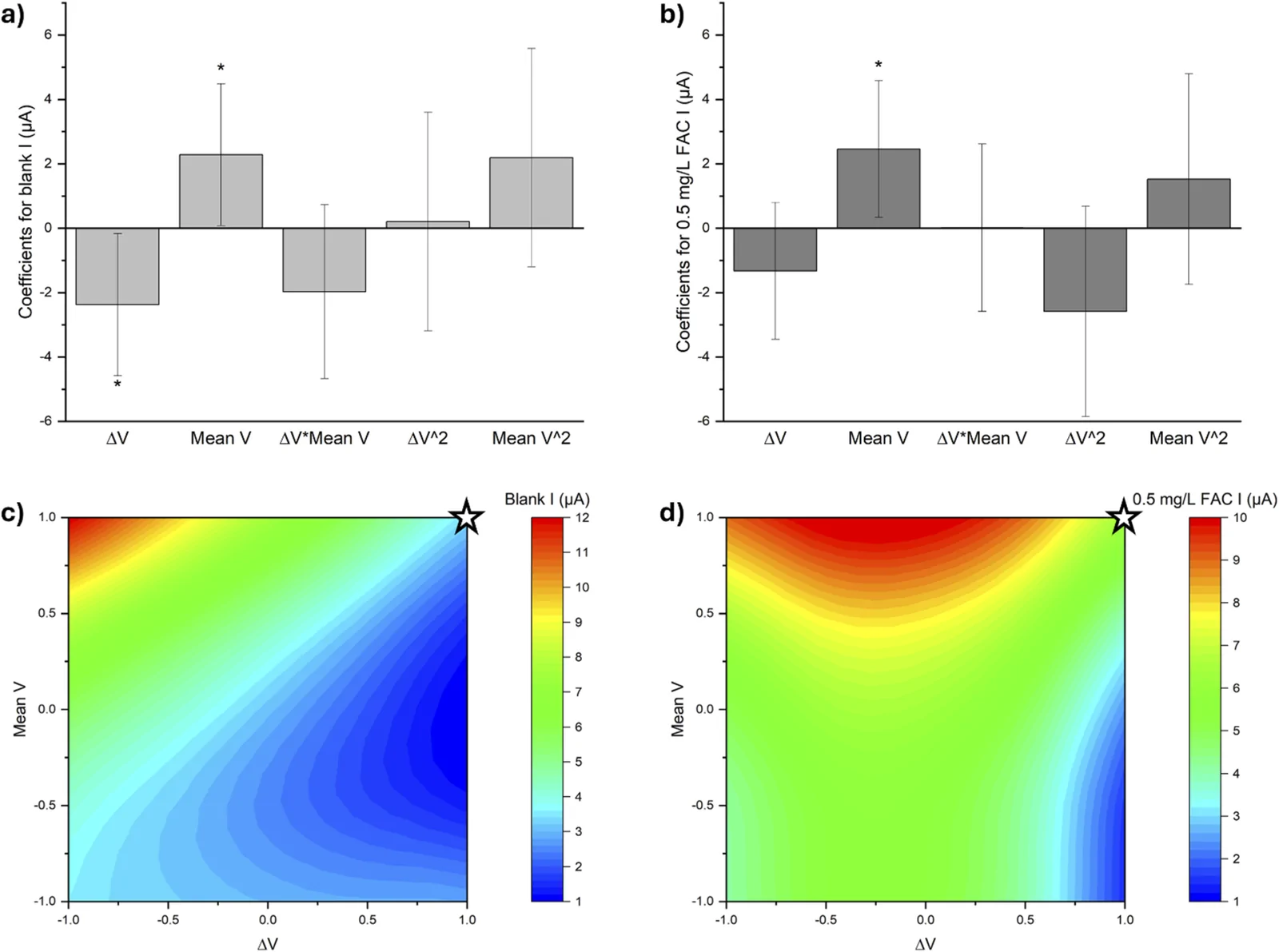
Coefficients plots (a and b) for *y*_1_, blank I (a) and *y*_2_, 0.5 mg L^−1^ FAC I, (b) computed by multiple linear regression, using fitting residuals for coefficients significance calculation (*: *p* = 0.05, **: *p* = 0.01, ***: *p* = 0.001) and response surfaces for the same responses (c and d).

The purpose of the optimization is to identify the amperometric settings that ensure the best compromise between low blank signal (*i.e.*, *y*_1_ minimization) and high FAC signal (*i.e.*, *y*_2_ maximization). From evaluation of the response surfaces (Fig. 2c and d), the best compromise is identified in the [1,1] conditions, where the blank current is predicted to be below 3 µA, while the analyte signal, for a fixed 0.5 mg L^−1^ FAC concentration, is around 7 µA. Considering the greatly reduced electrical noise achieved by replacing external cables with the microsoldered circuitry, this difference is considered satisfactory for our purposes; thus, all subsequent measurements are conducted by setting the potential difference to −100 mV the central potential value to 900 mV.

### 3.3 pH and conductivity-dependent calibration models development and evaluation

Once the optimal settings for FAC assessment are defined, the interference of pH and conductivity (*χ*) must be properly considered. The impact of pH on amperometric analyses is widely known in literature,^3,12,13,31^ and so is the interference of nitrates, sulfates and bicarbonates,^10^ which contribute to increasing overall matrix conductivity. Various strategies have been proposed in literature or in commercial devices to cope with this issue: lab-scale investigations rely on experimental plans in which these parameters are kept constant^10,31^ while commercial prototypes generally exploit more complicated, bulky and expensive setups^32^ or propose dedicated calibrations for different installation points. In this context, we propose general pH- and *χ*-dependent calibration models designed to be *in silico* tuned to the specific pH and *χ* values, measured in real time by the same NEMO device. The core idea behind this approach is that the current (*I*) value recorded during amperometric FAC determination depends not only on FAC concentration but also on pH and χ, and thus all these contributions must be taken into account.

To develop general calibration models, the experimental domain—defined as a function of the main features of interest, pH (6–8), *χ* (25–600 µS cm^−1^) and [FAC] (0–0.73 mg L^−1^) —is preliminarily mapped following a DoE-like rational sampling strategy, as described in Section 2.3.1. The overall list of calibration samples comprises 125 triplets of pH, *χ*, and [FAC], allowing a homogeneous and robust exploration of the domain, as depicted in Fig. 3a. The test set, instead (Fig. 3b) consists of four bottled-water samples for which pH and *χ* are not modified, and only FAC concentration is varied.

**Fig. 3:**
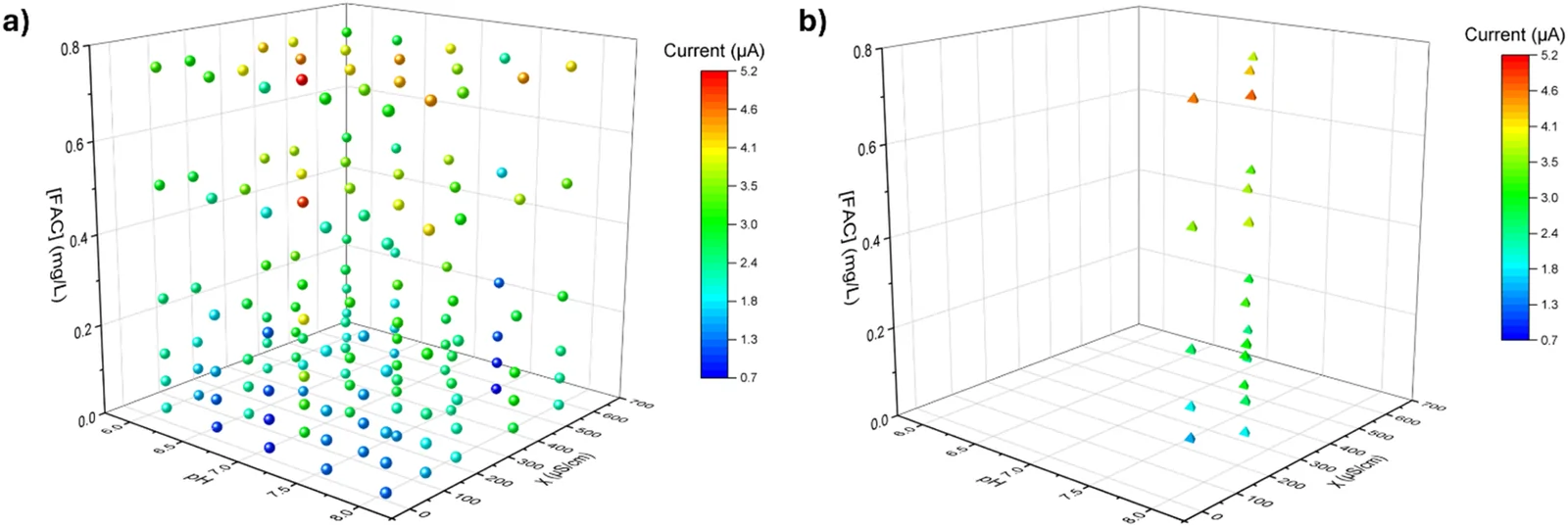
Calibration (a) and test samples (b) location in the experimental domain of interest.

Two approaches are then tested for calibration model development. Approach A relies on a multivariate equation (eqn (2)), developed using calibration samples as input data, for predicting the current (*I*) value for a given pH–*χ*–[FAC] triplet: the resulting I values are used to build specific calibration curves for every FAC measurement, based on the real-time measured values for pH and *χ* and on the FAC concentration levels used in the calibration set. The coefficients computed for the multivariate equation underlying Approach A are reported in Fig. 4a, together with their significance computed using the standard deviation of the fitting residuals. Several conclusions can be drawn from this plot: (i) the contribution of pH is negligible, indicating limited pH interference; (ii) the contribution of conductivity is instead significant, since both the linear and the quadratic term associated with this variable are consistent; (iii) the strongest effect is, as expected, associated with FAC concentration.

**Fig. 4:**
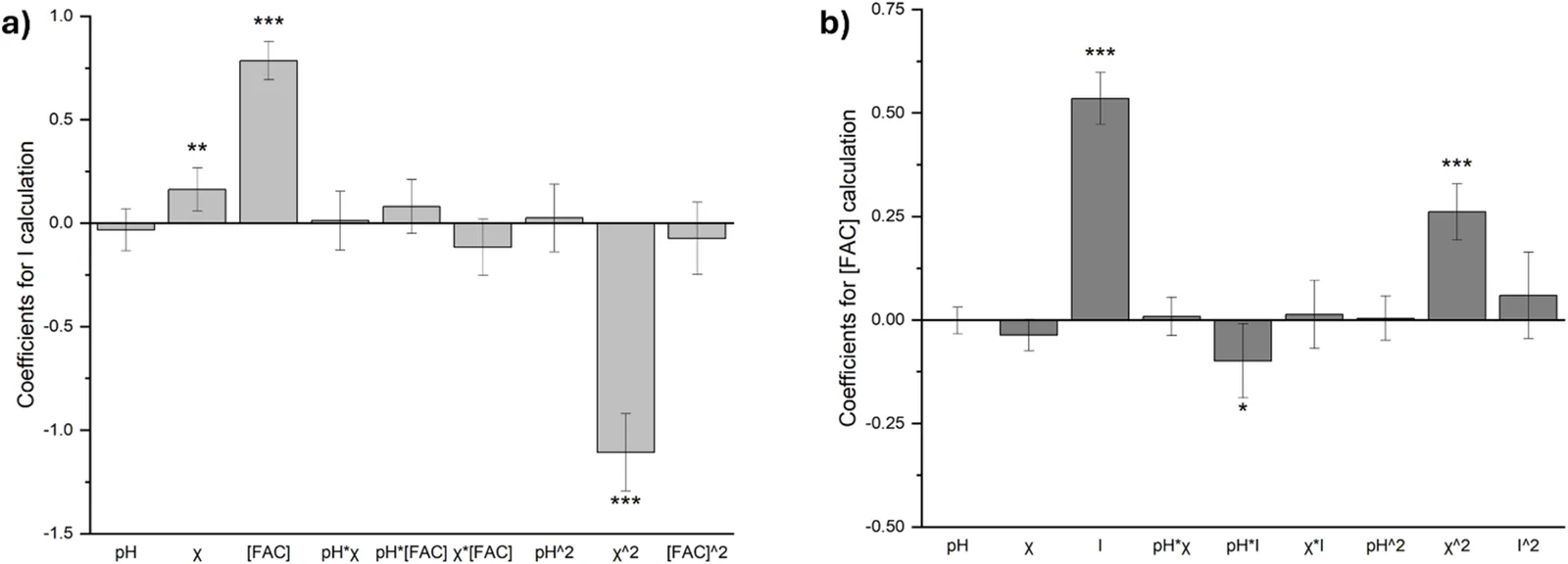
Coefficients computed for multivariate equations behind Approach A (a), referred to eqn (2), and B (b), referred to eqn (3).

Approach B is instead a single-step procedure in which calibration samples are used as input data to compute the model equation given in eqn (3) for direct FAC quantification from the pH–*χ*–I triplet. The contribution of the different parameters can be highlighted from the coefficient values in Fig. 4b: the current (*I*) value clearly represents the dominant effect, confirming a linear dependence between *I* and [FAC]. However, the quadratic effect of conductivity is also confirmed, together with the interaction between pH and current, found to significant at the 95% confidence level.

In providing a critical comparison between these two approaches, it is clear that both present advantages and limitations. Approach A ultimately provides a traditional calibration curve, even if developed *in silico*, thus allowing the use of standard statistical tools for analytical-method development. However, it presents three main limitations: (i) quadratic and interaction terms between input factors are included in the *I* calculation used in the *in silico* calibration, but are excluded from FAC calculation, increasing the risk of lack of fit; (ii) the considerably high number of degrees of freedom collected through the calibration samples (125 samples used to compute 10 coefficients) is not retained for FAC assessment, since the *in silico* calibration curves include only six FAC levels without replicates, and no variability is included; (iii) from a computational standpoint, integrating Approach A into the NEMO firmware is more challenging, as more steps are required for each measurement, including *in silico* simulation of the calibration procedure and curve calculation. Conversely, Approach B is computationally simpler, retains the calibration degrees of freedom, and includes higher-order terms in FAC calculation. However, it presents two main drawbacks: (i) well-known regression-line statistics cannot be directly applied, and less widespread or validated formulas must be derived for the multivariate model, as shown here for LOD estimation; (ii) the input variables are prone to multicollinearity, *i.e.*, being not fully independent and mutually correlated, a phenomenon that should be avoided when applying multivariate regression.

To assess the robustness of the regression coefficients and exclude instability due to correlated predictors, both multivariate calibration models (Approach A and Approach B) are evaluated through multicollinearity diagnostics. Variance Inflation Factors (VIFs) are computed for all linear, quadratic, and interaction terms included in the two MLR models. In all cases, VIF values remain well below commonly accepted thresholds for multicollinearity, with maximum values < 1.5 for both approaches. These results confirm that the predictors (pH, conductivity, current, and their interaction/quadratic terms) do not exhibit problematic collinearity and that the regression coefficients are statistically stable and interpretable. This also supports the suitability of Approach B, despite its more complex structure, and rules out the risk of inflated variances or unreliable parameter estimates.

Both models are preliminarily evaluated by comparing the errors associated with the multivariate models in cross-validation (leave-one-out approach) using predicted and experimental data for the test samples described in Table S3 and depicted in Fig. 3b. For Approach A, the comparison is made between I values, while the same procedure is applied directly to FAC values for Approach B. In both cases, the Root Mean Square Errors of Prediction (0.304 and 0.103, respectively) are lower than the equivalent values in cross-validation (0.556 for Approach A and 0.177 for Approach B), thus confirming the applicability of the models within the entire experimental domain.

A direct comparison between experimental and predicted values is presented in Fig. 5 for both approaches and target variables (*I* and FAC). The multivariate approach used for *in silico* calibration (Fig. 5a) shows good agreement between experimental and predicted *I* values, while the consequent FAC assessment (Fig. 5b) reveals a slight positive bias. Better results are obtained instead following Approach B (Fig. 5c), even though the semi-quantitative nature of the measurement is confirmed.

**Fig. 5:**
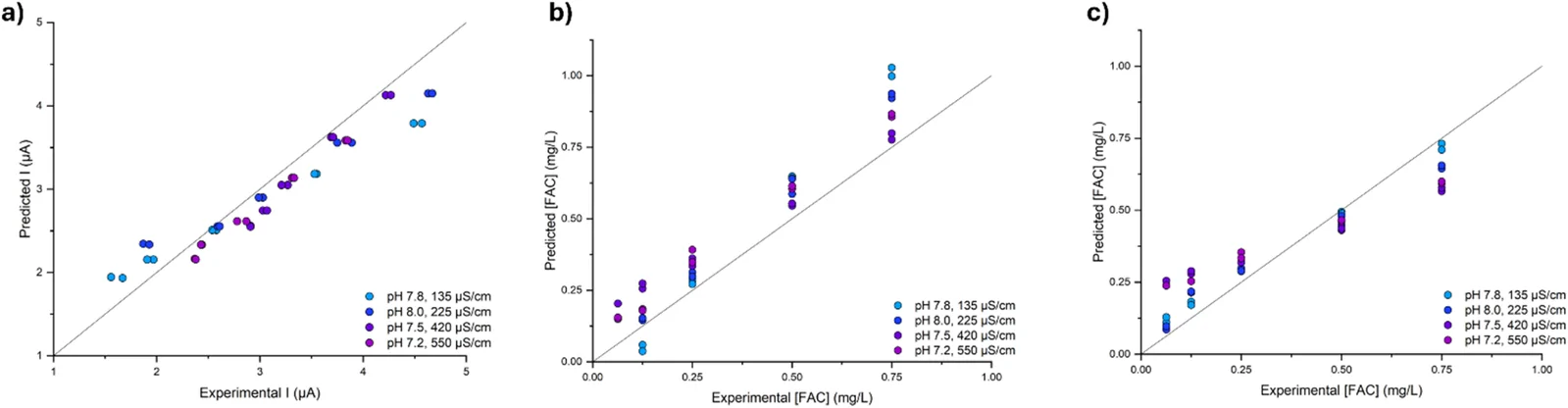
Experimental *vs.* predicted values for I (a) and consequent FAC (b) calculation according to Approach A and direct FAC calculation according to Approach B (c).

Although several multivariate calibration strategies have been proposed for electrochemical chlorine sensing, including Partial Least Squares (PLS), Support Vector Regression (SVR), and other nonlinear machine-learning approaches, their computational complexity makes them unsuitable for embedded, low-power OWQM devices.^16–18,21^ In NEMO, all processing must be executed directly on the microcontroller, without external computation or cloud resources, and with strict constraints on memory, battery consumption, and firmware size. Under these conditions, Multiple Linear Regression (MLR) represents the only fully implementable strategy: it requires a single closed-form evaluation of a polynomial equation, does not rely on iterative optimization, and avoids matrix decompositions or kernel transformations that would exceed the device's computational capabilities. This hardware-driven constraint motivated the selection of MLR over PLS or SVR. Therefore, while more sophisticated algorithms may theoretically achieve marginal gains, MLR provides the optimal trade-off between robustness, interpretability, and real-time deployability within the operational limits of NEMO.

### 3.4 Comparison with reference spectrophotometric method for approach A and B

A comparison between amperometric methods and the reference spectrophotometric procedure based on DPD reaction is established to assess the accuracy of Approaches A and B. DPD UV-Vis spectra are acquired for the pH and *χ* conditions used in the test set (B-E bottled waters in Table S3 or Fig. 3b) in the same FAC concentration range used for amperometric detection (0–0.75 mg L^−1^). Absorbance at 515 nm is extracted from UV-Vis spectra in Fig. 6a by means of a three-point baseline correction and used to compute matrix-dependent regression lines in Fig. 6b. As clearly visible, pH and *χ* show a significant effect in FAC determination by the DPD method, resulting in slightly different regression lines depending on the matrix composition.

**Fig. 6:**
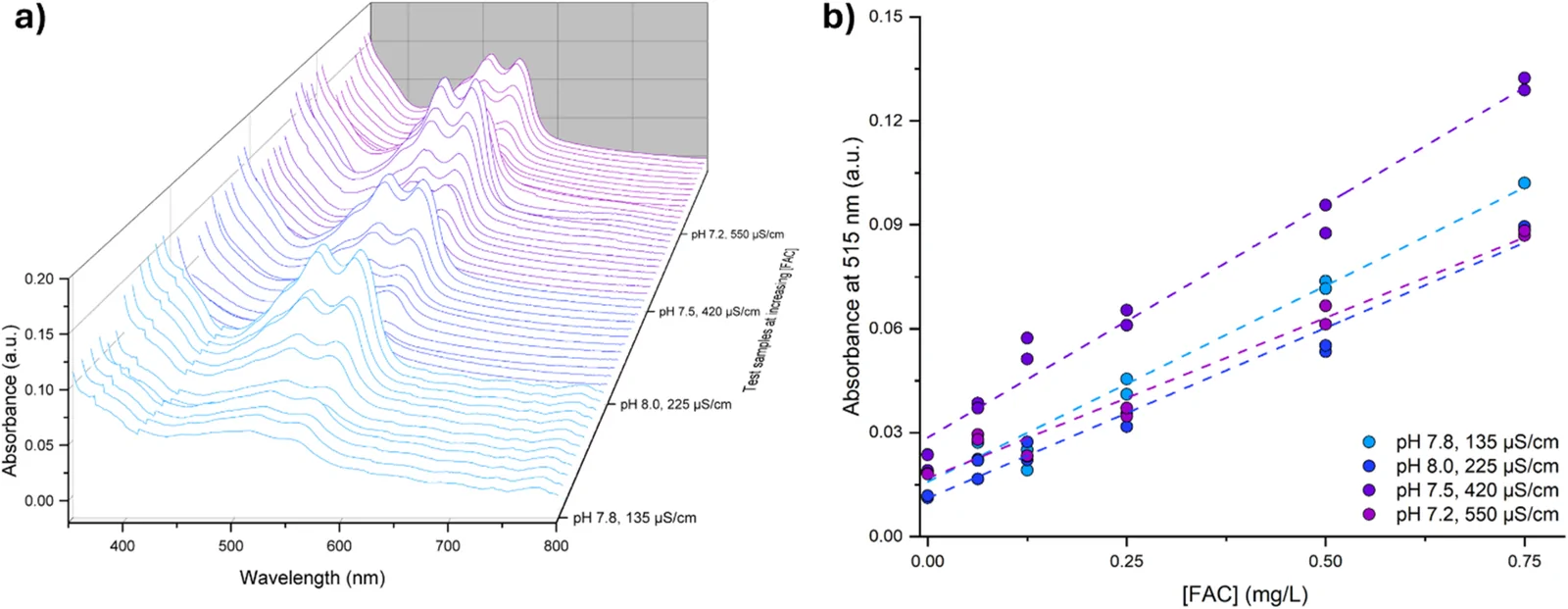
UV-Vis spectra (a) and regression lines (b) computed for test set conditions in 0–0.75 mg L^−1^ [FAC] in the case of DPD spectrophotometric method.

The resulting regression lines are used to calculate [FAC] in B-E bottled-water samples and to compare these results with [FAC] values assessed for the same samples by amperometric detection embedded in the NEMO device, using both Approaches A and B. The direct comparison is shown in Fig. S3a and b, and the residuals plots are shown in Fig. S3c and d. A two-ways ANOVA is applied to both comparisons (DPD *vs.* Approach A and DPD *vs.* Approach B) to assess the significance of matrix and method effects. When comparing Approach A with the reference DPD method, the matrix effect is significant at the 95% confidence level (*p*-value = 0.02, *), the interaction between matrix and method is significant at the 99% (*p*-value = 0.008, **), and the difference between the two methods is highly significant (*p*-value = 8 × 10^−6^, ***). In contrast, for Approach B, only the interaction between matrix and methods is marginally significant (*p*-value = 0.02, *), while neither the pH and *χ* conditions nor the quantification methodology are found to exert a significant impact on the result.

We can thus conclude that the amperometric method embedded in NEMO, coupled with the single-step Approach B for FAC quantification, ensures accuracy and robustness with respect to pH and *χ* conditions that are not significantly different from the spectrophotometric reference method, based on the DPD reaction.

### 3.5 LOD calculations according to procedure 1–4

Despite the semi-quantitative nature of the NEMO approach, having a rough idea of the LOD value is essential to evaluate the actual applicability of the device for in-field applications. In common analytical methods development, the LOD value can be computed using blank samples (Procedure 1 in Section 2.4.3), the standard deviation of calibration-sample residuals (Procedure 2) or following the iterative Haubax–Vos methodology, starting from blank values or residuals (Procedure 3 and 4, respectively). LOD definitions according to these four procedures are reported in the literature for traditional calibration lines and can be directly applied to Approach A while their extension to the multivariate calculations underlying Approach B is discussed in detail in Section 2.4.3.

Additionally, traditional calibration models built under specific matrix conditions (pH and *χ* in this case) are characterized by a single LOD value; however, in our case, the general models developed according to Approaches A and B are pH- and *χ*-dependent, and their mathematical equations change depending on matrix characteristics. For this reason, rather than a generalized LOD value, a specific pH- and *χ*-dependent value should be computed, to consider the dependence of model performance on these parameters.

Fig. 7 summarizes the LOD trend in the pH–*χ* domain under investigation for both the approaches and following different procedures. Several conclusions can be drawn from these plots: (i) the LOD value is generally around 0.2 mg L^−1^, with some extremely high values only in procedures relying on blank samples (Fig. 7a and b for Procedure 1 and Fig. 7e and f for Procedure 3); (ii) Procedure 2 (Fig. 7c and d), based only on calibration residuals, appears to show overly optimistic values across the entire experimental domain, while procedures based on blank values are more prone to variability, probably due to the limited number of blank samples measured. We can conclude that Procedure 4 (Fig. 7g and h) appears to be the most reliable procedure in this case, since it shows reasonable variations within the pH–*χ* domain.

**Fig. 7:**
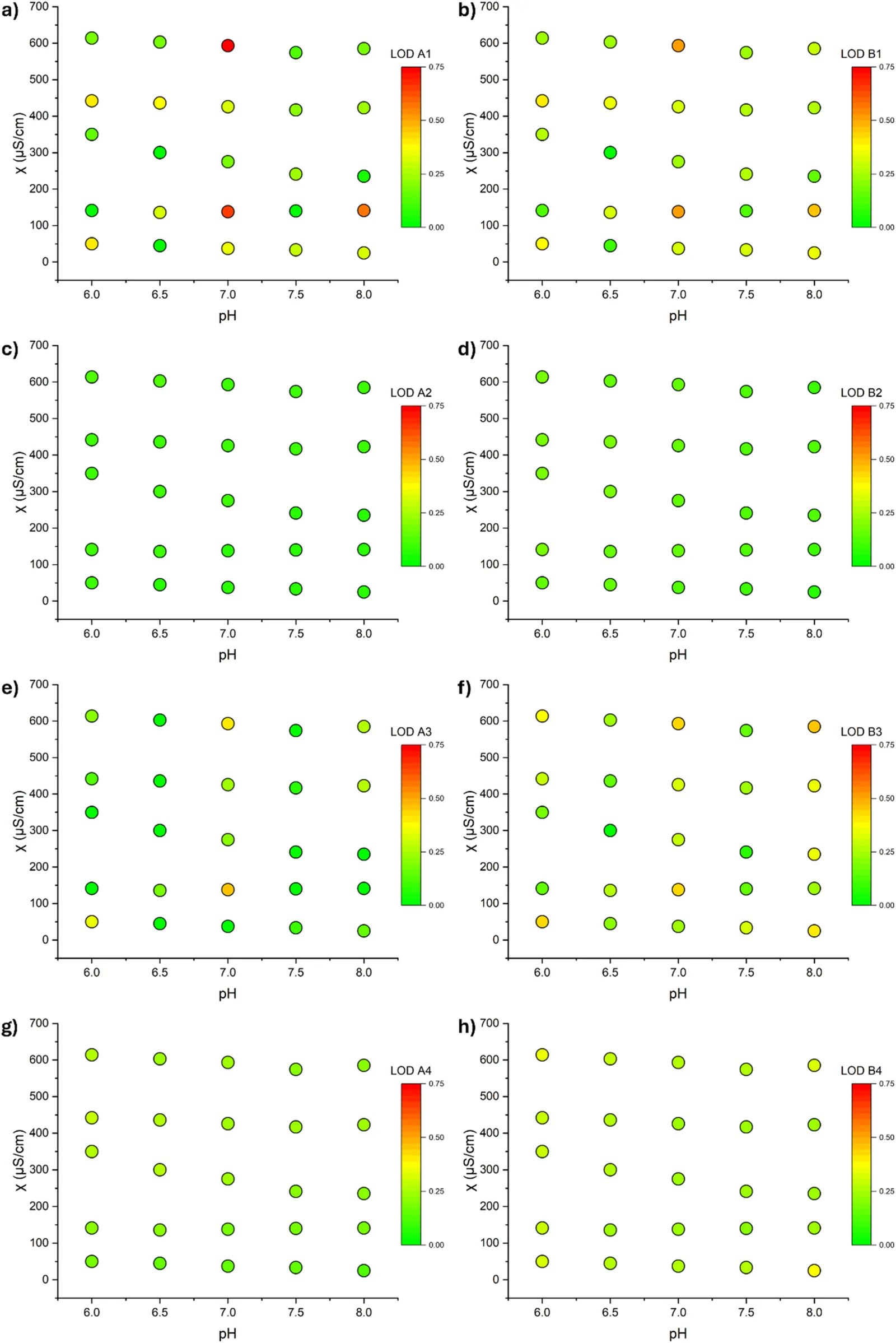
LOD values computed for FAC estimation using Approach A (a–d) and B (e–h) according to procedure 1 (a and e), 2 (b and f), 3 (c and g) and 4 (d and h).

It is worth noting that Procedures 1 and 3 rely on the experimental standard deviation of blank samples, and only two blank replicates were collected for each pH–conductivity combination. This limited sample size inevitably increases the variability of the corresponding LOD estimates, as these procedures are intrinsically sensitive to fluctuations in the measured blank signal. In contrast, Procedures 2 and 4 compute the significant signal from model-based quantities (predicted blank and residual standard deviation), which are derived from the full calibration dataset and therefore exhibit substantially higher numerical stability. For this reason, the LOD ranges obtained from Procedures 2 and 4 should be considered more robust and representative of the actual performance of the multivariate calibration models. We can thus conclude that the LOD value for the NEMO device is generally between 0.2 and 0.3 mg L^−1^; considering the final in-field application, such a range can be regarded as satisfactory.

A comparison with commercially available on-spot water quality monitoring devices was not included, as detailed and independently validated performance specifications for commercial FAC sensors are generally not publicly available. Most manufacturers report only marketing-level figures without disclosing calibration strategies, matrix-dependence, or long-term stability data, preventing a scientifically robust and reproducible benchmarking. For this reason, the present work focuses on fully transparent, experimentally validated performance metrics obtained under controlled laboratory conditions and in-field deployment.

### 3.6 Examples of NEMO device in field application

A preliminary evaluation of the applicability of NEMO devices for OWQM in WDSs is conducted by analysing the acquired pH, conductivity and I from six devices installed in a 6 km area in the Northern part of Italy during the period between May 8^th^, 2026, and July 6^th^, 2026 (Fig. 8a–c). The experimental triplets are then used as input values to compute FAC concentration according to both approaches previously described (Fig. 8d and e). As clearly visible from the plots in Fig. 8, the direct measurements of pH and conductivity are affected by considerable variability, especially in the first weeks after device installation. The amperometric signal shows greater robustness after installation, except for BS38 device.

**Fig. 8:**
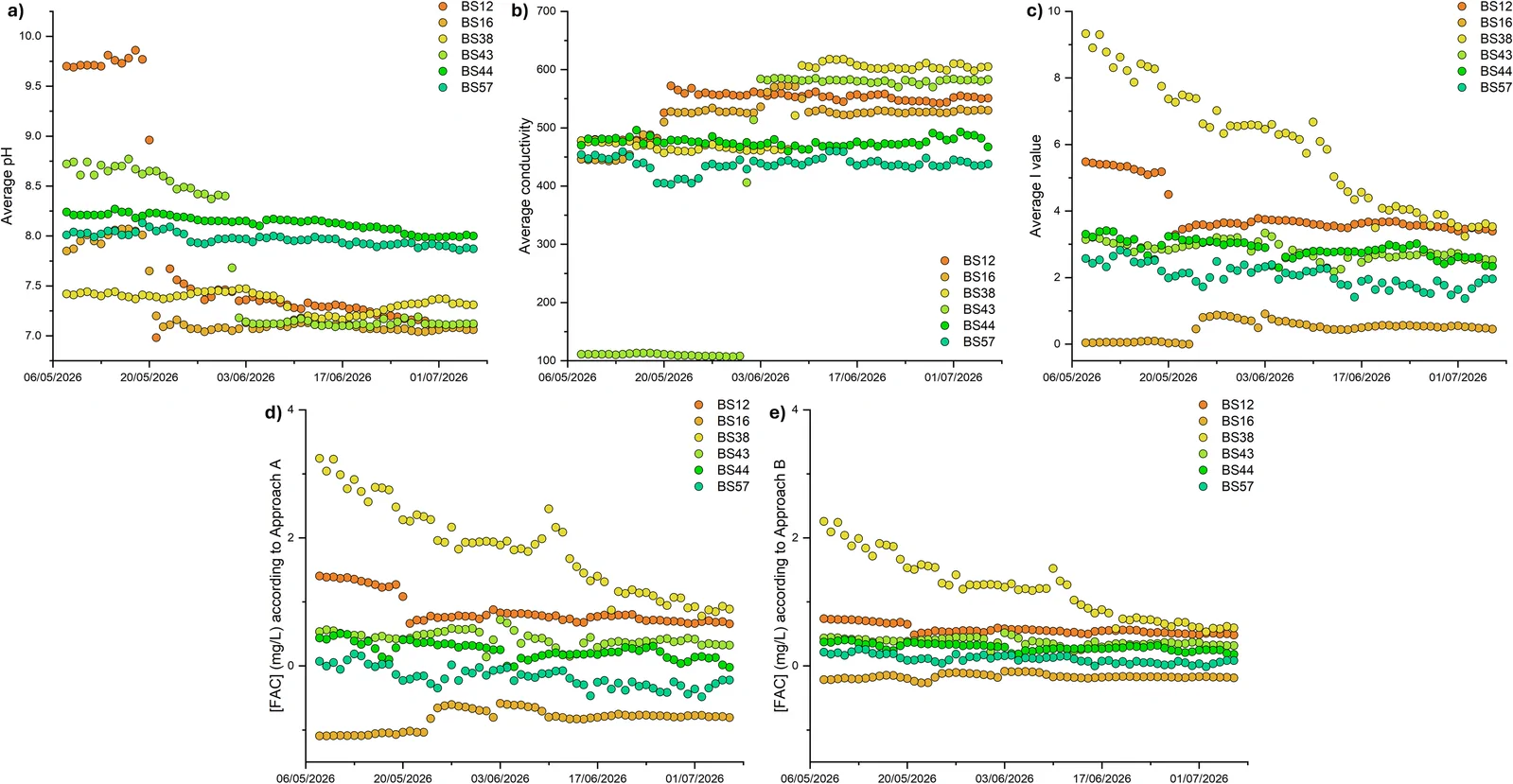
Acquired pH (a), conductivity in µS cm^−1^ (b) and *I* value in µA (c) and [FAC] in mg L^−1^ computed by the means of Approach A (d) and B (e) for in field applicability evaluation.

Finally, when comparing the two methods for FAC-concentration assessment, Approach A proves to be more sensitive but, on the other hand, more susceptible to experimental noise and parameter variability whereas Approach B shows increased robustness and less noisy results. The overall robustness for in-field application still needs to be improved, especially regarding calibration procedures and homogeneity across data acquired with different probes. A systematic field-validation against the DPD reference method is not feasible in this study due to logistical constraints associated with accessing the monitored distribution points and performing repeated manual sampling. Nevertheless, the FAC values provided by the local water utility for the monitored area consistently reported concentrations <0.5 mg L^−1^, in agreement with the range captured by the NEMO devices. The field experiment was therefore designed to assess signal stability, hysteresis behaviour, and the robustness of the embedded calibration models under real operating conditions, rather than to quantify absolute measurement accuracy.

## 4 Conclusions

This work demonstrates that reliable amperometric determination of FAC can be achieved using a low-cost, fully automated OWQM device, NEMO, specifically designed for continuous operation within real water-distribution systems. Through a multivariate optimization of the electrochemical parameters, we identified robust working conditions compatible with long-term field deployment and resilient to the intrinsic variability of tap-water matrices.

The development of pH- and conductivity-dependent calibration models, based on Multiple Linear Regression, successfully addressed one of the major limitations of amperometric chlorine sensing: the strong matrix dependence of the current signal. Both proposed strategies, an *in silico* calibration approach (Approach A) and a direct multivariate calibration model (Approach B), showed consistent predictive performance, with RMSEP values comparable to RMSECV and a consistent agreement with the reference DPD spectrophotometric method. The extensive evaluation of the limits of detection, carried out through four complementary procedures, confirmed that the models operate effectively within the concentration range relevant for drinking-water monitoring.

Field validation on six NEMO devices installed in a real distribution network over two months further confirmed the overall stability of the optimized protocol, a good level of reproducibility of the measurements, and the capability of the system to track temporal variations in FAC. The analysis also provided insight into hysteresis and long-term drift, two critical aspects for OWQM devices intended for early-warning applications.

Overall, this study shows that combining optimized electrochemical protocols, multivariate calibration strategies, and low-power embedded hardware enables consistent semi-quantitative monitoring of free chlorine in drinking water. The proposed methodology represents a practical and scalable solution for enhancing water-quality surveillance and supports the digital transformation of water-distribution infrastructures. While the proposed methodology provides a robust semi-quantitative estimation of FAC, we acknowledge that fully quantitative performance would require more sophisticated, power-intensive instrumentation and controlled operating conditions. Within the constraints of a compact, low-cost, battery-powered OWQM device, the semi-quantitative approach represents the most effective compromise between analytical performance and long-term field applicability, particularly for early-warning detection of anomalies in water-quality parameters. Future developments may extend this framework to additional chemical parameters, further broadening the analytical capabilities of distributed OWQM systems.

## Author contributions

Guglielmo Emanuele Franceschi: investigation, methodology, validation, formal analysis; Lisa Rita Magnaghi: conceptualization, data curation, visualization, writing – original draft, project administration; Gian Paolo Quarta: resources, funding acquisition, project administration, software; Raffaela Biesuz: supervision, writing – review and editing.

## Conflicts of interest

There are no conflicts to declare.

## Abbreviations

FAC: Free available chlorine
WDS: Water distribution systems
OWQM: Online water quality monitoring
EWS: Early warning system
DPD: *N*,*N*-Diethyl-*p*-phenylenediamine
CV: Cyclic voltammetry
WE: Working electrode
RE: Reference electrode
CE: Counter electrode
*χ*
: Conductivity
DoE: Design of experiments
MLR: Multiple linear regression
RMSEP: Root mean square error of prediction
RMSECV: Root mean square error in cross-validation
LOD: Limit of detection
UL_b_: Upper limit of the blank confidence interval
LL_LOD_: Lower limit of the LOD confidence interval
SDGs: Sustainable development goals
EDTA: Ethylenediaminetetraacetic acid

## Supplementary Material

RA-016-D6RA07165A-s001

## Data Availability

Raw data supporting this study cannot be made available due to commercial restrictions. Supplementary information (SI) is available. See DOI: https://doi.org/10.1039/d6ra07165a.
