# Supplementary material for: A multivariate approach to amperometric free chlorine monitoring: calibration, interference compensation, and application in water distribution systems
Source: RSC Adv. 2026 Sep 25;16(45):57427–41. doi: 10.1039/d6ra07165a (PMC13613367; doi:10.1039/d6ra07165a)
Supplement: Supplementary file 1 [file RA-016-D6RA07165A-s001.pdf]

Supplementary Information (SI) for RSC Advances.
This journal is © The Royal Society of Chemistry

A Multivariate Approach to Amperometric Free Chlorine Monitoring: Calibration, Interference Compensation, and Application in Water Distribution Systems

Guglielmo Emanuele Franceschi[a], Lisa Rita Magnaghi*[a,b], Gian Paolo Quarta[c] and Raffaela Biesuz[a,b]

[a] *Department of Chemistry, University of Pavia, Viale Taramelli 12, Pavia (PV), 27100, Italy*

[b] *INSTM, Unità di Ricerca di Pavia, Via G. Giusti 9, Firenze (FI), 50121, Italy*

[c] *Onyax – Soluzioni IoT, Via P. Bertolini, 9/L, Vigevano (PV), 27029, Italy*

Supplementary Material

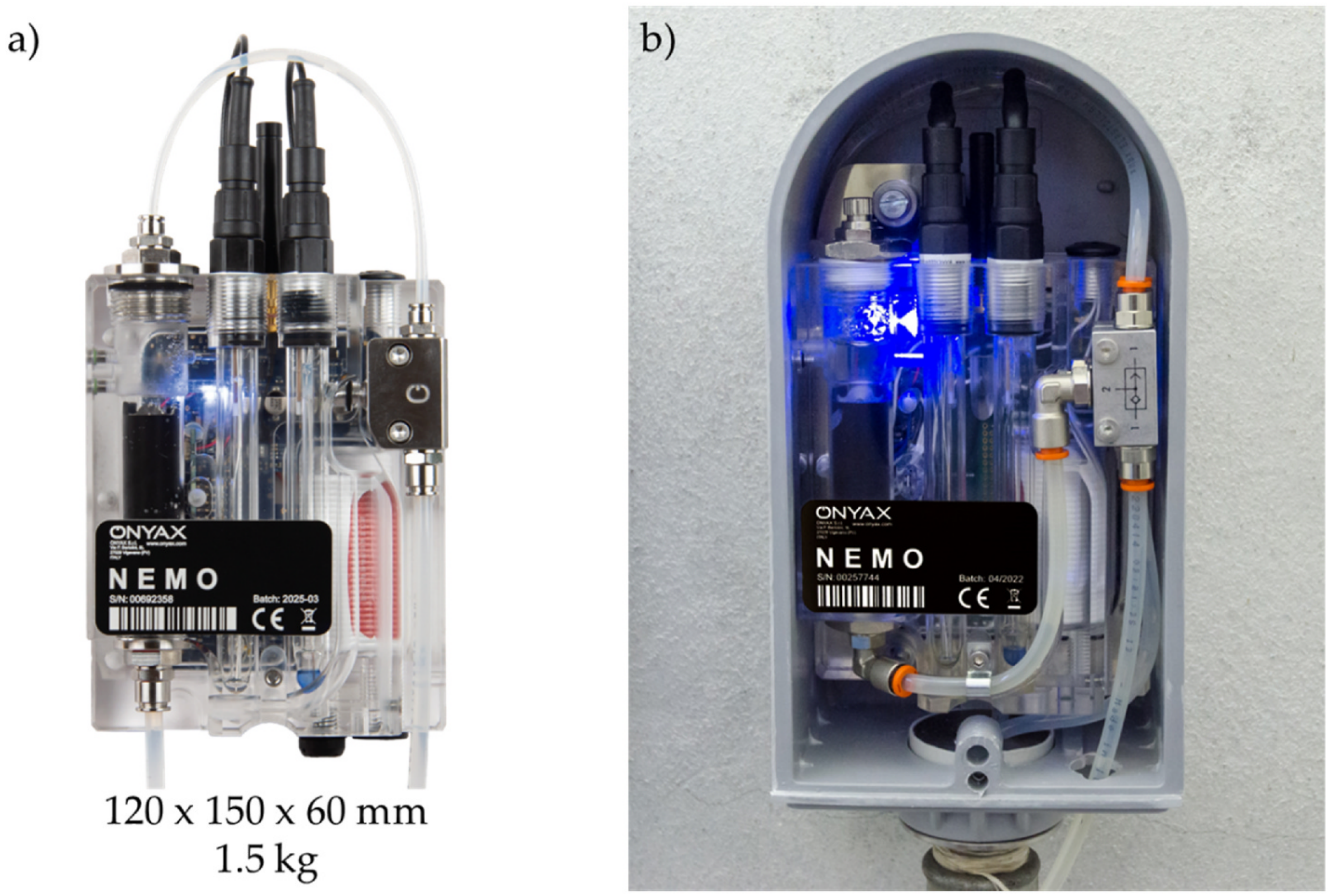

Figure S1: a) OWQM device NEMO, developed and produced by Onyax srl; b) NEMO installation in distribution system's road shell for OWQM.

Table S1: Preparation details for Ciclyc Voltammetry (CV) analysis

N	pH	Buffer	Buffer concentration (M)	[FAC] (mg/L)
1	3.5	Citrate buffer	0.01	0
2	3.5	Citrate buffer	0.01	2.5
3	3.5	Citrate buffer	0.01	5.0
4	5.5	Citrate buffer	0.01	0
5	5.5	Citrate buffer	0.01	2.5
6	5.5	Citrate buffer	0.01	5.0
7	7.5	Phosphate buffer	0.0025	0
8	7.5	Phosphate buffer	0.0025	2.5
9	7.5	Phosphate buffer	0.0025	5.0
10	9.5	Borate buffer	0.0025	0
11	9.5	Borate buffer	0.0025	2.5
12	9.5	Borate buffer	0.0025	5.0

Table S2: Experimental matrix and plan for settings optimization by DoE, together with responses values

N	x_1	x_2	∆V	V	y_1	y_2
1	-1	-1	-300 mV	500 mV	4.67	5.93
2	1	-1	-100 mV	500 mV	1.42	2.61
3	-1	1	-300 mV	900 mV	12.75	8.84
4	1	1	-100 mV	900 mV	1.61	6.24
5	0	-1	-200 mV	500 mV	3.28	2.37
6	0	1	-200 mV	900 mV	8.70	11.22
7	-1	0	-300 mV	700 mV	3.95	3.39
8	1	0	-100 mV	700 mV	4.07	2.00
9	0	0	-200 mV	700 mV	3.01	7.71
10	0	0	-200 mV	700 mV	2.19	6.23
11	0	0	-200 mV	700 mV	2.60	6.97

Table S3: Test samples for pH and conductivity-dependent calibration

Bottled water	Actual pH	Actual χ
B	7.8	135 μS/cm
C	8.0	225 μS/cm
D	7.5	420 μS/cm
E	7.2	550 μS/cm

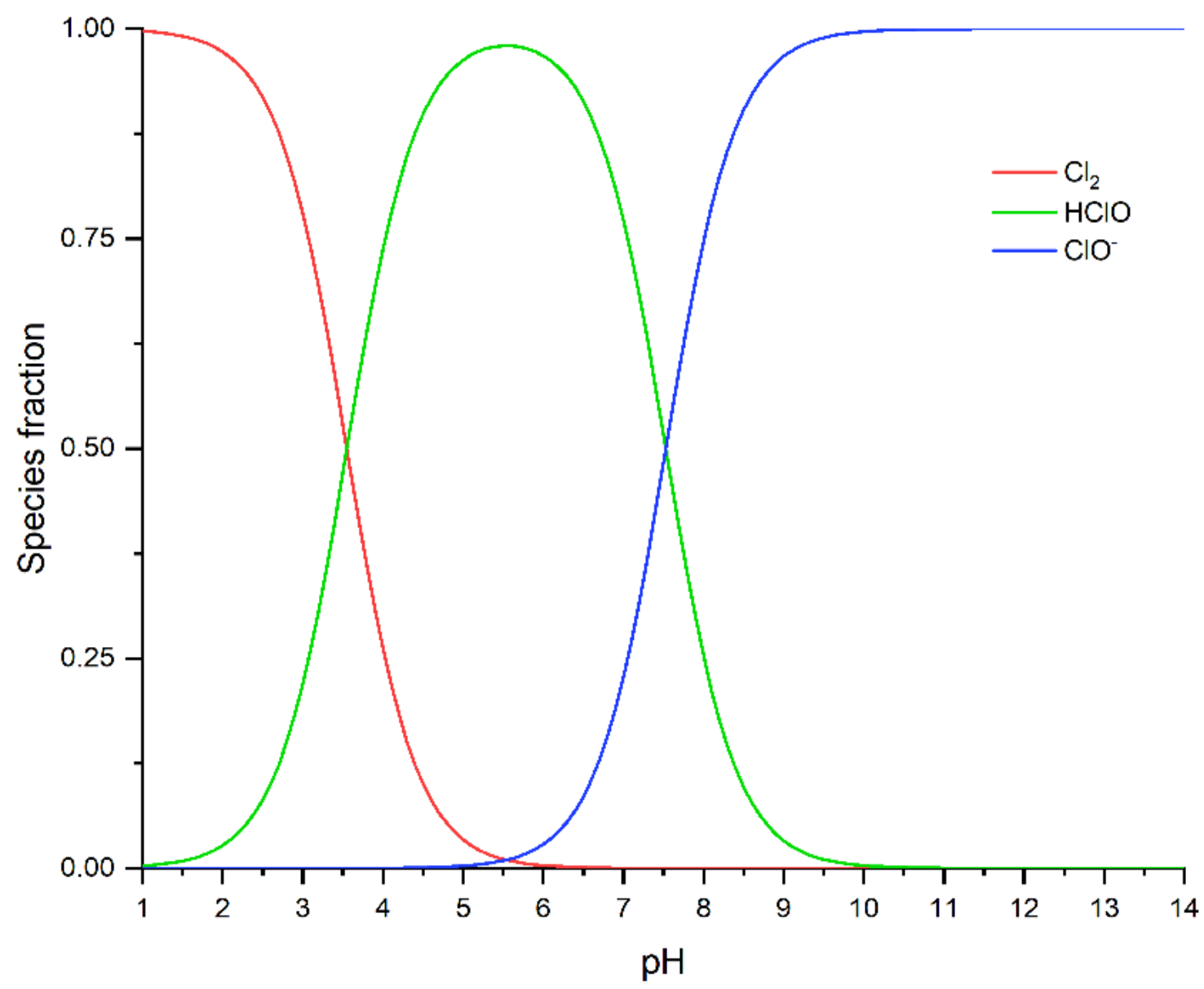

Figure S2: Speciation equilibrium for chlorine-related species

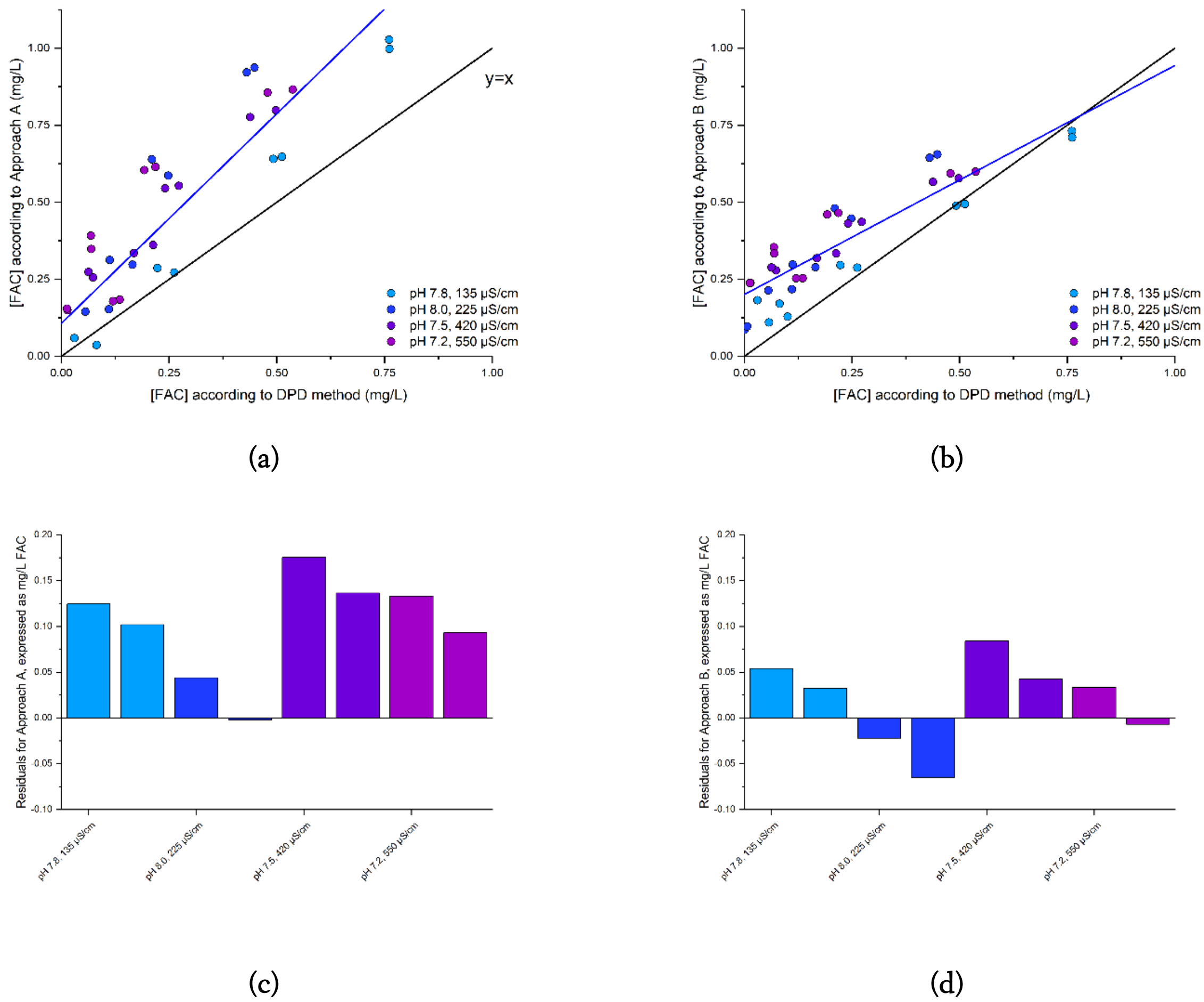

Figure S3: Comparison between FAC concentration calculated by DPD spectrophotometric method vs. amperometric detection coupled with approach A (a) and B (b) and corresponding residuals (c and d for Approach A and B, respectively) in the case of test samples in Table S3

Table S4: Face-centred design models statistics for fitting and Leave-One-Out CV.

Bottled water	Blank-sample current (y_1)	0.5 mg/L FAC current (y_2)
Residuals SD	2.099	1.976
% E.V.	62.2%	54.7%
RMSECV	4.451	3.924
